# Dissolved phosphorus transport from soil to surface water in catchments with different land use

**DOI:** 10.1007/s13280-014-0617-5

**Published:** 2015-02-15

**Authors:** Dries Verheyen, Nele Van Gaelen, Benedicta Ronchi, Okke Batelaan, Eric Struyf, Gerard Govers, Roel Merckx, Jan Diels

**Affiliations:** 1 Department of Earth and Environmental Sciences, Celestijnenlaan 200E-2411, 3001 Leuven, Belgium; 2 Department of Earth and Environmental Sciences, Celestijnenlaan 200E-2410, 3001 Leuven, Belgium; 3 Department of Earth Sciences, Flinders University, GPO Box 2100, Adelaide, SA 5001 Australia; 4 Department of Biology, University of Antwerp, Universiteitsplein 1, 2610 Wilrijk, Belgium; 5 Department of Earth and Environmental Sciences, Celestijnenlaan 200E-2409, 3001 Leuven, Belgium; 6 Department of Earth and Environmental Sciences, Kasteelpark Arenberg 20-2459, 3001 Leuven, Belgium

**Keywords:** Dissolved P, Land use, Headwater catchment, Pathways

## Abstract

Diffuse phosphorus (P) export from agricultural land to surface waters is a significant environmental problem. It is critical to determine the natural background P losses from diffuse sources, but their identification and quantification is difficult. In this study, three headwater catchments with differing land use (arable, pasture and forest) were monitored for 3 years to quantify exports of dissolved (<0.45 µm) reactive P and total dissolved P. Mean total P exports from the arable catchment ranged between 0.08 and 0.28 kg ha^−1^ year^−1^. Compared with the reference condition (forest), arable land and pasture exported up to 11-fold more dissolved P. The contribution of dissolved (<0.45 µm) unreactive P was low to negligible in every catchment. Agricultural practices can exert large pressures on surface waters that are controlled by hydrological factors. Adapting policy to cope with these factors is needed for lowering these pressures in the future.

## Introduction

Phosphorus (P) is the main element responsible for eutrophication of surface waters in Europe, and enrichment of aquatic ecosystems by P originating from anthropogenic sources has become a huge environmental issue (e.g. Csatho et al. 2007; Kronvang et al. 2007). In the past 20 years, Europe has discharged 0.2–0.3 Tg P year^−1^ to its coastal waters (Grizzetti et al. 2012). These high exports of P can be ascribed to excessive use of fertiliser and manure, resulting in a P imbalance in agricultural soils in developed countries (Vitousek et al. 2009). The imbalance is exacerbated by having large numbers of livestock in a limited area and importing their feed, since the high cost of off-farm manure disposal leads to excessive organic P fertilisation on agricultural land in areas with intensive livestock production (Hooda et al. 2001).

The main losses of P from the anthropogenic environment to surface waters thus originate from agricultural land (e.g. Sharpley et al. 2000; Cordell et al. 2009). Struyf et al. (2012) found a twofold increase in P concentration in the sediment of the Berg River, South Africa, under increasing human influence. Phosphorus is a non-renewable resource and therefore P losses are detrimental to the long-term sustainability of agriculture. According to Childers et al. (2011), the anthropogenic P cycle on agricultural land must be closed, so that no losses to the environment occur. Losses of P due to human activities other than agriculture can also be very significant (Smil 2000) and there are major challenges associated with reducing the legacy P built up in previous decades (Sharpley et al. 2013).

Fertilisation recommendations and current legislation are mainly aimed at controlling the P content in soil (Tunney et al. 2003). Easily measurable factors such as ammonium lactate-extractable P (P-AL) or degree of phosphorus saturation (DPS) (van der Zee 1988) are generally used to determine the potential of P to reach surface waters. Official determination of permissible P levels is most often based on crop nutrient requirements (P-AL) or the P binding capacity of the soil. However, different soils can have differing susceptibility to P losses, which cannot be measured with soil P tests (Sharpley et al. 2000). Hence soil type, in combination with catchment hydrology, can greatly affect the risk of P reaching aquatic environments. A number of previous studies have attempted to explicitly account for hydrological factors in risk assessment (e.g. Sharpley et al. 2003a; Heathwaite et al. 2005). The three main pathways through which mobilised P can reach surface waters are surface runoff, subsurface flow and vertical flow to the groundwater–surface water interaction zone. The pathway activated depends on rainfall pattern and duration and the interval between rainfall events (Haygarth et al. 2000). Natural and artificial drainage characteristics also play a key role. The highest risk of P transport to a water body arises when a significant source of P has good hydrological connectivity to surface waters. Such areas are known as critical source areas (Sharpley et al. 2003b).

Phosphorus transport from soil to surface waters occurs in different forms. Particulate P (PP) is the most important fraction during storm runoff events (Heathwaite and Dils 2000; McDowell et al. 2001; Steegen et al. 2001). Particulate P losses are highly related to the level of sediment erosion in a catchment, which depends on catchment morphology, vegetation and land use (Steegen et al. 2001). During base flow, concentrations are usually low, with the dominant form of exported P being reactive P (RP; <0.45 µm) originating from groundwater sources (Haygarth et al. 2005). High concentrations of reactive P during base flow are mostly related to incidental P losses, e.g. from cleaning events (Haygarth et al. 2004).

Dissolved organic P (DOP) is reported to play an important role in P transport within soils, via the soil matrix and preferential flow paths (Chardon et al. 1997; Turner and Haygarth 2000). As DOP is more mobile than orthophosphate in soils (Turner and Haygarth 2000), it may also reach surface waters more quickly. However, little information exists concerning the role of DOP and dissolved (<0.45 µm) P associated with inorganic colloids in overall P transport at the landscape scale (e.g. Stamm et al. 1998; Hens and Merckx 2002). This obviously hinders assessment of the effect of human activity on P dynamics and export under agricultural land use. While total P TP (>0.45 µm) associated with mobilised soil clearly is a major source of P losses, a full understanding of the possible impact of management interventions can only be achieved if all P sources are accounted for. Furthermore, it is important that water quality goals are set correctly, which requires P export under undisturbed conditions to be correctly understood and quantified (Delgado and Scalenghe 2008). At present, policy norms are set based on the little information available and thus there is a risk that such norms may be simply unattainable.

This study examined the dynamics of dissolved (<0.45 µm) P in three headwater catchments with a similar climate but under different land uses (forest, pasture and arable) in three consecutive years. Export processes and transport pathways of different chemical forms of dissolved P to the catchment outlet were compared for the three catchments. Phosphorus concentrations were monitored in stream water, soil water and groundwater. Since all three catchments are headwater catchments not affected by industrial or household waste, P exports must originate from diffuse P sources. Our starting hypothesis was that human activities elevate P export from headwater catchments and that the P loss pathways differ greatly between land uses due to management and vegetation cover differences. The 3-year dataset also allowed seasonal variations in P transport pathways to be investigated.

## Materials and methods

### Study area

**Fig. 1:**
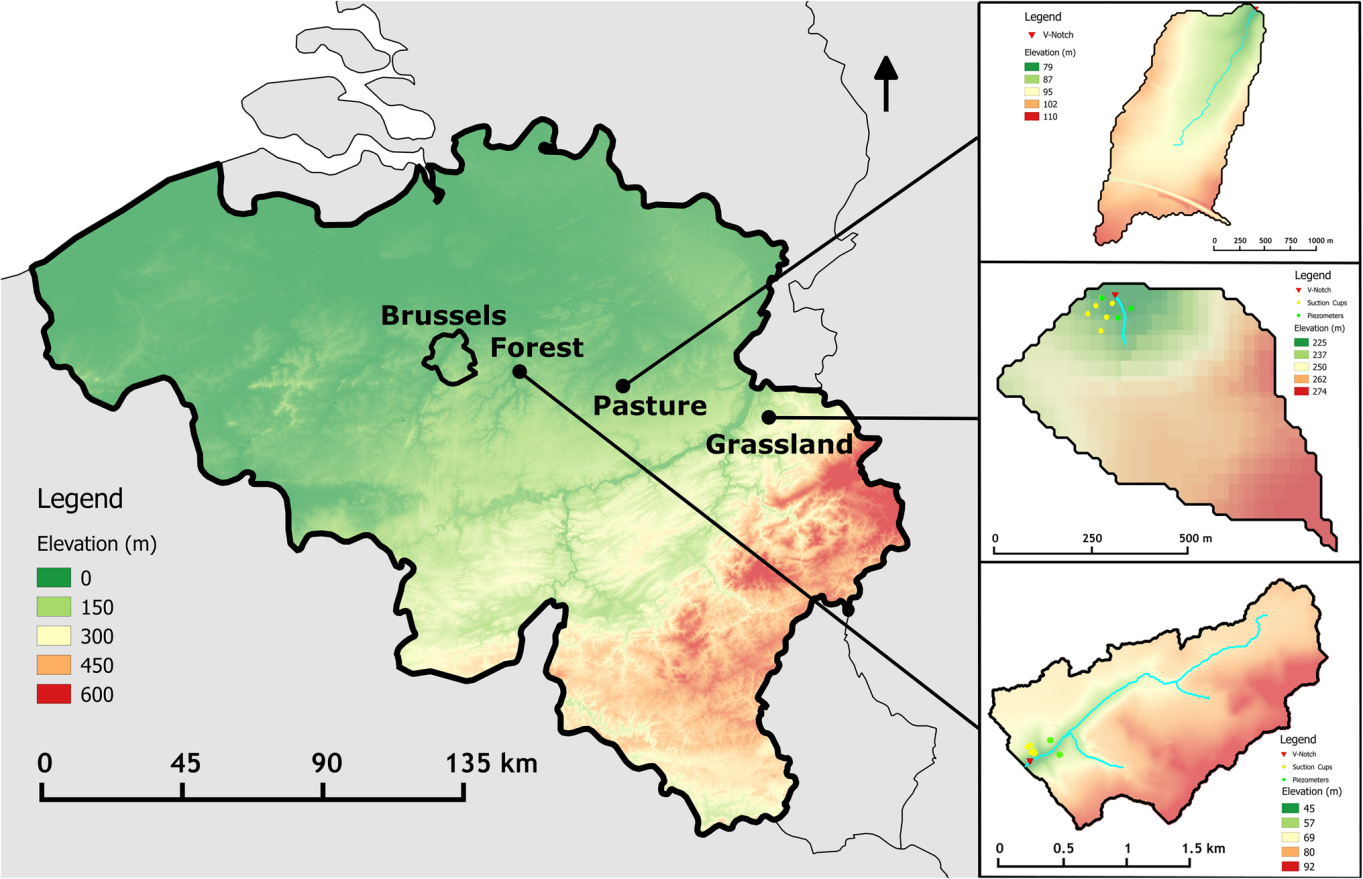
Overview of the location of the different catchments with elevation map of Belgium as background. Catchment elevation maps are given in detail with the position of the samplers (*red* *triangles*), suction cups (*yellow* *circles*) and piezometers (*green* *circles*)

All three headwater catchments are located in the Belgian loam belt (Fig. 1). The climate is temperate, with long-term mean precipitation of 760 mm year^−1^ and mean monthly temperature of 3.1 °C in January and 17.7 °C in July. The precipitation is uniformly distributed over the year, but varies in intensity between seasons. Summer rainfall events are usually of short duration but more intense (up to 60 mm h^−1^), while winter rainfall events are prolonged and less intense (generally <10 mm h^−1^).

The 2.33 km^2^ arable catchment (5°7′26″E, 50°45′36″N) is located in the village of Velm and is the upstream catchment of the Heulen Gracht, a tributary of the Molenbeek river. The elevation varies between 65 and 110 m above sea level (a.s.l.). An aeolian loam layer of 2–4 m was deposited on this relief during the last glaciation period and this loamy layer covers the Hannut tertiary formation, which contains mostly sand and some sandstone layers. The land use is mainly arable (90 %), while the rest of the area is occupied by apple orchards and some pasture, mostly on steeper slopes. The catchment suffers severe soil erosion from the arable land during intense rainfall. Grass strips and transverse earthen walls have been constructed in the thalweg of the Heulen Gracht catchment in order to trap eroded sediment.

The 0.34 km^2^ pasture catchment (5°45′52″E, 50°40′23″N) is located in Blegny, an area with somewhat steeper topography (gradient up to 20 %), where the elevation varies between 200 and 280 m a.s.l. The catchment is located in the headwater stream of the Lhonneux river. Geologically, it is characterised by the chalk layers of the Gulpen formation (Cretaceous). These are covered by silex-conglomerate, which in turn is covered with a stone-rich clay layer (Barchy and Marion 2000).

The 2.66 km^2^ forest catchment (4°40′12″E, 50°48′2″N) is situated in Sint–Joris–Weert and is under deciduous forest, with mixed oak (*Quercus robur* L) and beech (*Fagus sylvatica* L.) stands. The study area is the upstream part of the Paddenpoel river, where the elevation varies between 50 and 95 m a.s.l., and slope gradients reach 25 %. An aeolian loamy layer of the Pleistocene covers the Tertiary deposits of Brussels sands in this catchment. In these permeable sands, the groundwater level is at approximately 12 m depth under the hilltops and sandy outcrops with podsol formation can be found (Peeters 2010). The topography is undulating, so the groundwater depth varies and groundwater discharge occurs near the outlet of the catchment.

### Field measurements

Discharge of the streams in the pasture and forest catchments was measured with a V-notch weir, while the arable catchment was gauged with a San Dimas flume. Water height (m) was automatically measured every 15 min with a flow module (ISCO 710 Ultrasonic module or ISCO 720 submerged vented pressure transducer module, Teledyne ISCO Inc., Lincoln NE, USA) connected to an automatic water sampler and data logger (ISCO 6712, Teledyne ISCO Inc., Lincoln NE, USA). From the water height measurements, discharge (m^3^s^−1^) was calculated. In all three catchments, precipitation amount and intensity were measured using a tipping bucket rain gauge. In the forest catchment, precipitation measurements took place in an open field near the forest, at 1.04 km distance from the V-notch weir.

In all catchments, sampling was carried out from January 2011 to December 2013. In this 3-year period, flow samples (800 mL) were collected from base flow and peak flow regimes in the river by the ISCO sampler. Base flow was automatically sampled twice a week, while the peak flow samples were automatically taken in a flow-proportional manner. A maximum of 15 flow samples could be taken per peak flow event. The samples were collected from the sampler within 3 days of a peak event, or within a maximum of 2 weeks if no peak flow was detected. Samples were stored in the ISCO sampler in polyethylene terephthalate (PET) bottles and no refrigeration or heating was used on-site.

To obtain samples of soil water, suction cups (plastic, polyamide; Eijkelkamp, Giesbeek, The Netherlands) were placed in every catchment in two parallel transects perpendicular to the river channel a year before sample collection, to allow them to equilibrate with their environment. The transects started close (<10 m) to the river and the suction cups were spaced between 15 and 45 m apart at three points along every transect, at three different depths (30, 60 and 90 cm) at every point. Samples of soil water were taken monthly by applying continuous suction of about 500 hPa using a vacuum pump (Irrometer Soil Sampling Vacuum Pump, Eijkelkamp, Giesbeek, The Netherlands). Groundwater monitoring and sampling were carried out by installing a single piezometer at three different locations in the forest and pasture catchment but not in the arable catchment, where the deep groundwater does not contribute to stream flow. Groundwater samples were taken monthly with a peristaltic pump (12 Vdc, Eijkelkamp, Giesbeek, The Netherlands). Groundwater depth was measured manually (as a check) and automatically every 30 min using unvented pressure transducers (Mini-Diver^®^, Eijkelkamp, Giesbeek, The Netherlands). The readings were post-processed for on-site measured barometric pressure.

In the pasture catchment, three seeps were found to contribute water to the stream all year round. These seeps are perennial seepage faces arising from groundwater intersecting with the slope at one side of the river close to the stream. Monthly grab samples were taken from those seeps.

### Data collection

Approximately the same number of samples was taken in all three catchments (Table 1). However, a lower number of groundwater samples and seep samples were taken in the pasture catchment in 2011 because sampling only started in June for the groundwater samples and September for the seep samples. Two extra piezometers were installed in November 2012 in the pasture catchment and no gaps occurred in collection of rainfall and discharge data. In the forest catchment, there were some gaps in discharge data due to battery failure or malfunctioning of the equipment. However, in most cases, a water sample was still taken, so that no significant gaps occurred in the measurement of concentrations (Table 2).

**Table 1:** Number of samples taken in each catchment according to year and type of sample

	Year	Forest	Pasture	Arable
Streamwater base flow	2011	156	86	–
	2012	86	85	–
	2013	83	80	–
Streamwater peak flow	2011	163	110	68
	2012	43	110	79
	2013	61	51	63
Soil water	2011	76	59	14
	2012	66	93	57
	2013	33	70	14
Groundwater	2011	38	8	–
	2012	36	13	–
	2013	26	33	–
Seeps	2011	–	18	–
	2012	–	47	–
	2013	–	39	–

**Table 2:** Properties of the three catchments in each study year. The completeness values are the available data (% of the period) after removal of sensor errors and battery failures

	Year	Forest	Pasture	Arable
Completeness discharge (%)	2011	72.8	99.9	99.9
	2012	56.4	99.5	99.1
	2013	75.7	100.0	97.9
Completeness rainfall (%)	2011	100.0	100.0	100.0
	2012	99.9	99.9	100.0
	2013	98.5	100.0	100.0
Precipitation (mm)	2011	557.0	549.4	610.0
	2012	586.6	734.5	756.0
	2013	517.6	739.2	609.0
Stream discharge (mm)	2011	2.2	168.7	37.9
	2012	2.5	135.1	22.1
	2013	1.9	195.9	18.4
TP (<0.45) export (kg year^−1^)	2011	0.4	3.9	51.5
	2012	0.6	2.6	23.6
	2013	0.5	3.4	22.9
Areal TP (<0.45) export (kg ha^−1^ year^−1^)	2011	0.0014	0.12	0.22
	2012	0.0022	0.08	0.10
	2013	0.0019	0.10	0.10
Flow-weighted mean TP (<0.45) concentration (mg L^−1^)	2011	0.067	0.069	0.59
	2012	0.088	0.058	0.47
	2013	0.099	0.051	0.54
Area (ha)		265.6	34.0	229.9

### Chemical analysis

Prior to analysis, all samples collected in the field were stored at 4 °C in darkness, as recommended by Worsfold et al. (2005). Upon arrival at the laboratory or otherwise within 1 week of collection, a subsample of each sample was filtered through a 0.45 µm CHROMAFIL© cellulose mixed ester membrane filter. These subsamples were stored in polyethylene bottles at 4 °C in darkness. As soon as possible, but within 6 weeks at most, the samples were analysed. All the analyses were performed on the <0.45 µm fraction of the samples, so only dissolved P was determined. All the P terminology used in this paper follows the suggestions of Haygarth and Sharpley (2000). The time between collection from the field and filtering was kept as short as possible to reduce the risk of bacteria changing the P speciation. This could otherwise have lowered the RP (<0.45) concentration after storage, resulting in overestimation of UP (<0.45).

Two types of P measurements were carried out to evaluate differences in P speciation between samples. First, RP (<0.45) was measured with a colorimetric method (Van Veldhoven and Mannaerts 1987). This ion association method uses formation of a green-coloured ion association complex of 12-molybdophosphoric acid (12-MPA) with malachite green oxalate after acidification of the sample to pH 0 with 3 M H_2_SO_4_. The absorbance of the solution at 630 nm is then measured, here using a spectrophotometer (Perkin-Elmer Lambda 20, Waltham MA, USA) with 1 cm path length. With this method, orthophosphate together with the colloidal P fraction and some organic P fractions is measured (Van Moorleghem et al. 2011). The detection limit is 0.02 mg P L^−1^. It should be noted that the acid environment of the chemical reaction (pH 0–1) can cause hydrolysis of the organic P or displacement of P from colloids in the sample (Baldwin 1998). Second, total P (TP) (<0.45) and concentrations of Na, Mg, K, Ca, S, Al, Cu, Fe and Mn (mg L^−1^) were measured by ICP-OES (Optima 3300 DV, Perkin Elmer Waltham MA, USA). Phosphorus was measured after acidification of the samples to pH 1 with a 5 M HNO_3_ solution. Unreactive P (UP) (<0.45) was then calculated as the difference between TP (<0.45) and RP (<0.45). Unreactive P (<0.45) can also be considered as the dissolved organic P (DOP) fraction.

## Results

### Phosphorus analysis

Both techniques used for measuring P (<0.45) detected almost the same concentration of P (data not shown). This suggests that the samples taken in these catchments contained very little dissolved, unreactive organic fractions of P, although it is possible that the UP (<0.45) fractions of the samples were easily hydrolysable and were therefore also measured with the colorimetric reaction. At low TP (<0.45) concentrations, however, some samples contained much higher RP (<0.45) concentrations relative to TP. This was most likely due to the presence of a relatively high concentration of DOC interfering with spectroscopic measurement of the colorimetric reaction by colouring the sample. This effect can be expected to be proportionately larger at low concentrations of P. In groundwater and soil water, RP (<0.45) and TP (<0.45) concentrations were usually very low (<0.2 mg L^−1^). At such low concentrations, UP (<0.45) is difficult to quantify with certainty.

### Dissolved phosphorus budgets

Rainfall was normal in all three catchments during all three study years (Table 3). In the arable catchment, the highest yearly discharge was not observed in the wetter year 2012 (22.1 mm) but in 2011 (37.9 mm). This was because more of the intense rainfall events in 2011 took place when the soil was susceptible to surface sealing and runoff. Runoff peaks in the summer in the arable catchment occurred very sporadically when conditions were such that rainfall intensity exceeded soil infiltration capacity. During winter, soil saturation resulted in excess runoff. During such runoff events, average TP (<0.45) concentrations were high (0.34–0.60 mg P L^−1^) (Table 3), with a yearly flow-weighted mean TP (<0.45) concentration of 0.47–0.59 mg P L^−1^ (Table 2). In the pasture catchment, TP (<0.45) concentrations were generally an order of magnitude lower (0.05–0.07 mg P L^−1^), but total annual TP (<0.45) exports from the pasture catchment were almost the same as those from the arable catchment. This was because the total yearly runoff was an order of magnitude higher, as the stream had a constant base flow. Flow-weighted mean annual TP (<0.45) concentrations in the forest catchment were similar to those measured in the pasture catchment. This was because the stream runoff in the forest catchment was very low compared with the rainfall. The drainage area of the outlet of the forest catchment, calculated from the elevation map, is much larger than the groundwater catchment area. Piezometer measurements indicated that the groundwater level south of the stream is lower than the stream water level, so the stream only receives groundwater flow from one side of the catchment. This leads to very low base flow, while peak flow discharge in the stream can be several hundred-fold greater than the base flow when a rainfall event occurs. Even though the flow-weighted mean TP (<0.45) concentrations measured in the forest catchment were comparable to those measured in the pasture catchment, the total areal P flux out of the forest catchment was two orders of magnitude lower (1.4–2.2 g P ha^−1^ year^−1^; Table 2), due to the small runoff quantities.

**Table 3:** Mean and range of TP (<0.45) concentration (mg L^−1^) in different water samples from the three catchments in each study year. Standard deviations are given in brackets

			Forest	Pasture	Arable
Streamwater base flow	2011	Mean	0.17 (±0.13)	0.04 (±0.05)	–
	2012	Mean	0.19 (±0.12)	0.06 (±0.04)	–
	2013	Mean	0.09 (±0.08)	0.05 (±0.03)	–
Streamwater peak flow	2011	Mean	0.08 (±0.04)	0.12 (±0.16)	0.60 (±0.10)
		Range	0.024–0.31	0.004–0.44	0.36–0.75
	2012	Mean	0.10 (±0.06)	0.09 (±0.03)	0.34 (±0.19)
		Range	0.021–0.28	0.022–0.21	0.089–0.77
	2013	Mean	0.07 (±0.03)	0.12 (±0.07)	0.59 (±0.23)
		Range	0.026–0.18	0.002–0.33	0.24–1.05
Soil water	2011	Mean	0.08 (±0.26)	0.06 (±0.07)	0.07 (±0.002)
	2012	Mean	0.01 (±0.02)	0.07 (±0.26)	0.03 (±0.03)
	2013	Mean	0.01 (±0.01)	0.06 (±0.12)	0.02 (±0.01)
Groundwater	2011	Mean	0.03 (±0.04)	0.02 (±0.03)	–
	2012	Mean	0.02 (±0.01)	0.09 (±0.15)	–
	2013	Mean	0.02 (±0.01)	0.04 (±0.03)	–
Seeps	2011	Mean	–	0.08 (±0.05)	–
	2012	Mean	–	0.06 (±0.02)	–
	2013	Mean	–	0.04 (±0.03)	–

A striking observation was that the mean base flow concentration of TP (<0.45) was higher than the mean peak flow concentration in the forest catchment, while the reverse was observed in the pasture catchment. This suggests that dilution occurs in forest, while in pasture an important P source is activated during peak flows, transporting a higher concentration of P to the stream. In all three catchments studied, anticlockwise hysteresis was always observed during runoff events (data not shown). In the beginning of the peak event in the arable catchment, the concentrations of TP (<0.45) quickly increased together with the discharge, reflecting runoff sources of P being activated during the event. After the peak, concentrations of TP (<0.45) continued to be high and constant, reflecting constant delivery or dissolution of TP (<0.45) from stream-bed sediment into the stream. The variation in discharge was more pronounced than the variation in TP (<0.45) concentration. Anticlockwise hysteresis in the forest catchment was caused by dilution of the P concentration during peak events. The size of the hysteresis loop in the pasture catchment was small and less pronounced.

Soil water, groundwater and seep (pasture catchment only) samples did not contain high concentrations of TP (<0.45) in any of the catchments and no clear temporal pattern was observed. On average, soil water concentrations were low in all catchments during the 3-year study period, but sometimes highly variable (0.05 ± 0.16 mg P L^−1^) and occasionally reached 0.77 mg P L^−1^ in the pasture catchment. Groundwater concentrations were stable and low all year round in all catchments (0.03 ± 0.06 mg P L^−1^). Similarly, average TP (<0.45) concentrations in the seeps in the pasture catchment were low (0.05 ± 0.03 mg P L^−1^).

### Seasonal, event and base flow fluctuations in phosphorus

**Fig. 2:**
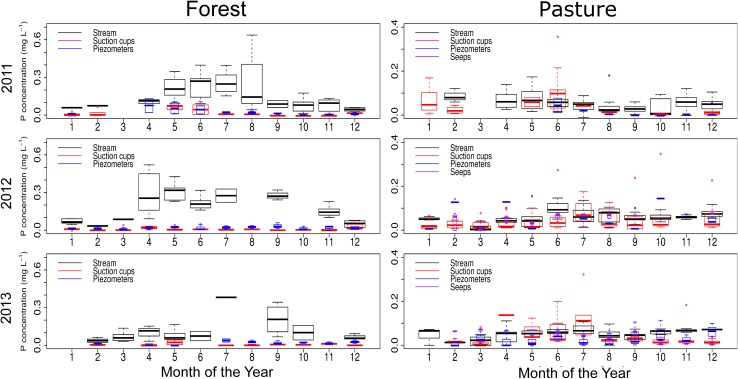
Boxplots showing the variation in TP (<0.45) concentrations during base flow conditions in stream water, suction cups, piezometers and seeps over the year. Boxplots show median and 25th and 75th percentiles and whiskers 10th and 90th percentiles

In the forest catchment, the base flow TP (<0.45) concentration showed a clear seasonal variation (Fig. 2). In winter months (October to March), the concentration was low, between 0.01 and 0.18 mg P L^−1^. The concentration rose in the summer months (April to September) up to 0.64 mg P L^−1^. The seasonal pattern was similar to that of air temperature, especially in 2011 and 2012. A clear seasonal pattern was not observed in the groundwater and soil water, where concentrations were low all year round. However, in the beginning of spring, the concentration occasionally rose in soil water samples, due to the warmer temperatures in the soil enhancing P mineralisation. This means that there must be another source of P entering the stream in summer.

Unlike the forest catchment, the base flow concentrations in the pasture catchment were low and nearly constant over the year (Fig. 2). During the 3-year measurement period, base flow in the pasture catchment fluctuated seasonally. It was low in November, but in December it rose quickly to its highest value, after which it slowly decreased again. The stream reacted quickly to rainfall events that caused the discharge to rise quickly. After the rain, discharge quickly decreased back to pre-event level.

**Fig. 3:**
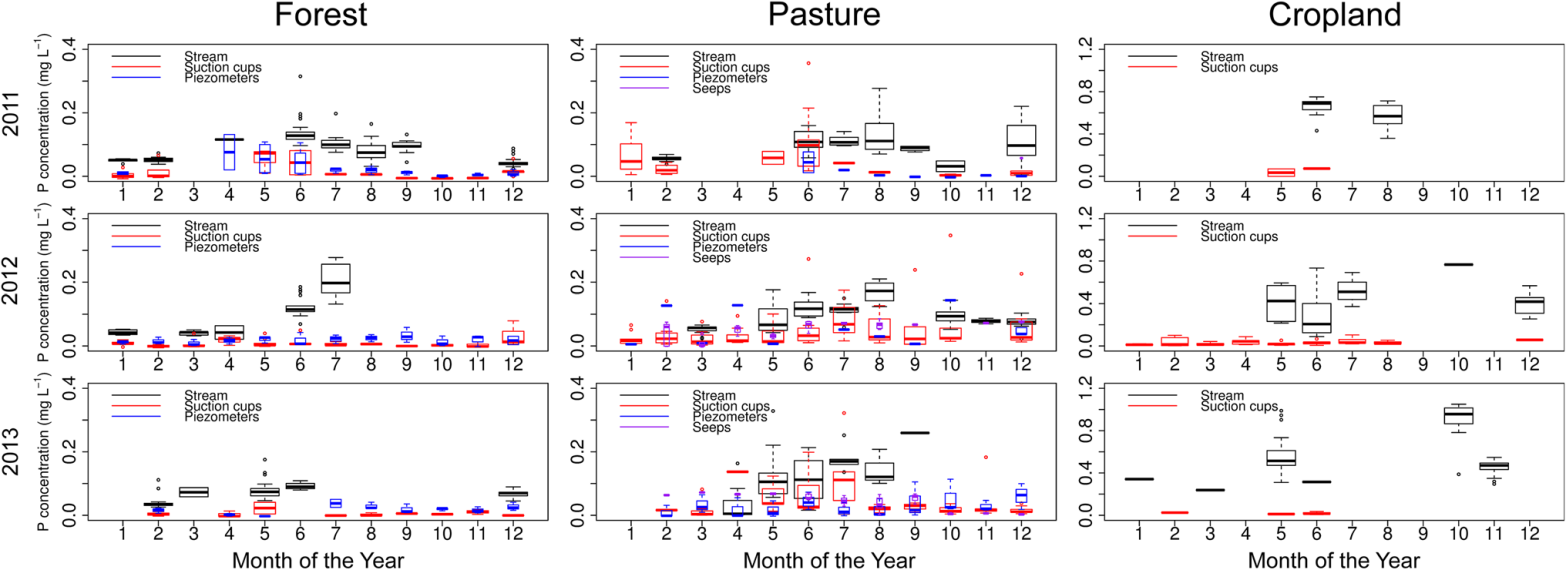
Boxplots showing the variation in TP (<0.45) concentrations during peak flow conditions in stream water, suction cups, piezometers and seeps over the year. Boxplots show median and 25th and 75th percentile and whiskers 10th and 90th percentiles

**Fig. 4:**
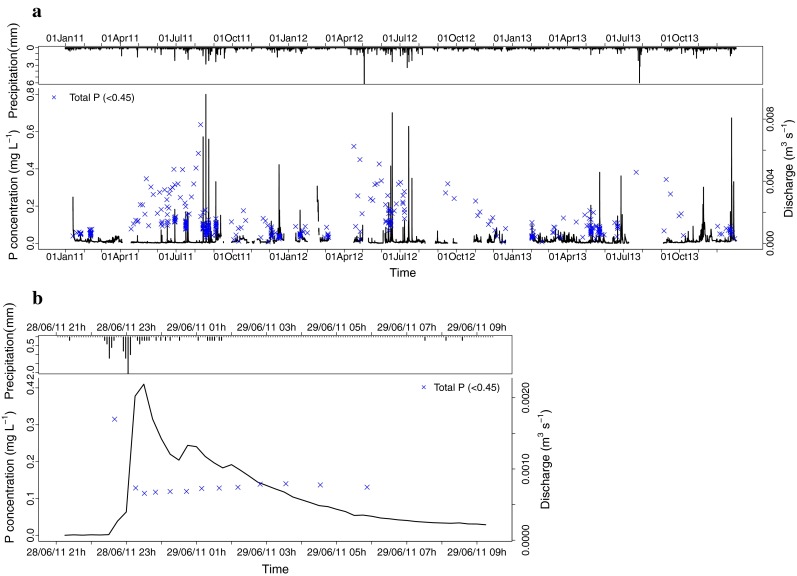
Precipitation, discharge and TP (<0.45) concentration in the forest catchment during **a** the three-year study period and **b** a detailed peak event on 28–29 June 2011

**Fig. 5:**
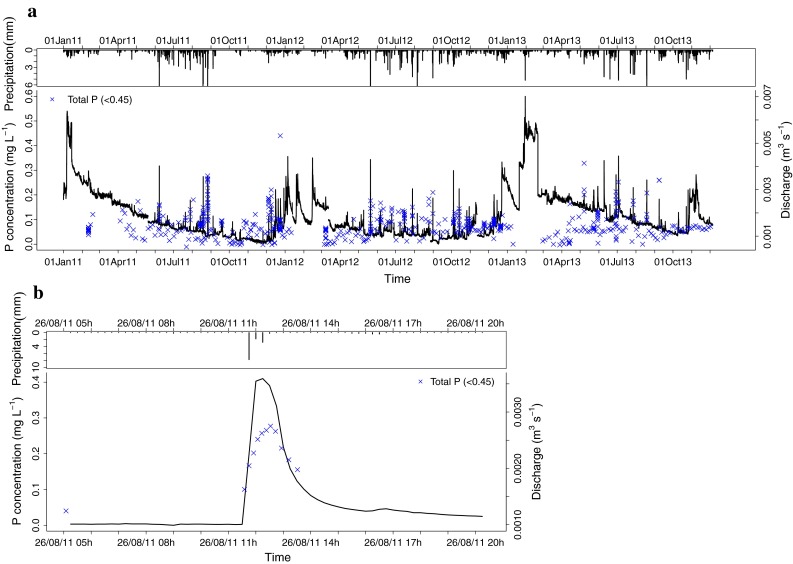
Precipitation, discharge and TP (<0.45) concentration in the pasture catchment during **a** the 3-year study period and **b** a detailed peak event on 26 August 2011

In the forest catchment, peak concentrations of TP (<0.45) were higher in summer months than in winter months (Fig. 3). Before peak discharge events, there were higher concentrations of TP (<0.45), while the base flow discharge was low. When a rainfall event started, the TP concentration dropped and discharge rose rapidly. During the peak, the concentration remained almost the same, while in the falling limb of the discharge, TP (<0.45) concentration tended to rise, but only slightly. This process was apparent in almost all the peak measurements taken in the forest catchment (Fig. 4a). In the pasture catchment too, the increase in peak flow P concentrations in summer was mostly larger than the discharge decrease, leading to higher P export in summer than in winter. The TP (<0.45) concentration rose and fell together with the discharge (Fig. 5b). The peak of TP (<0.45) generally occurred slightly later than the discharge peak.

**Fig. 6:**
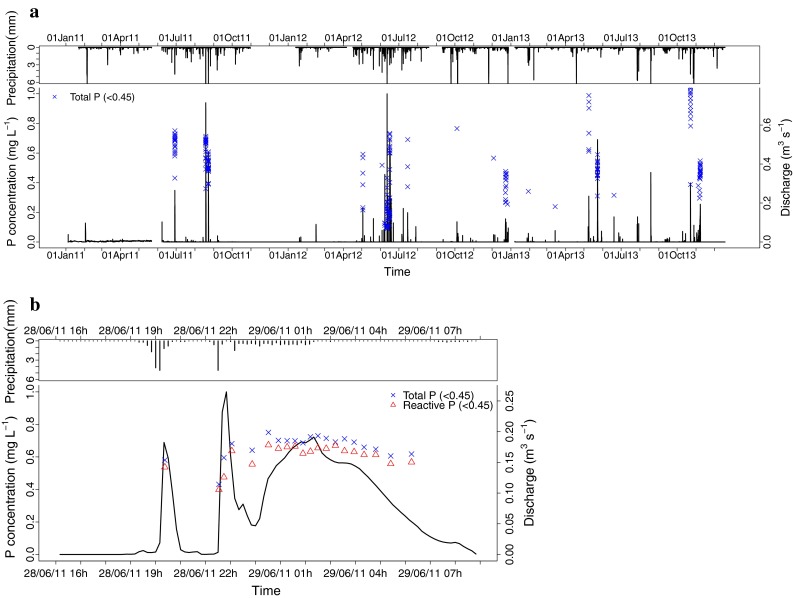
Precipitation, discharge and TP (<0.45) concentration in the arable catchment during **a** the 3-year study period and **b** a detailed peak event on 28 June 2011. The RP (<0.45) concentration is also given in (**b**)

In the arable catchment, the P concentration was high and nearly constant during the whole duration of a runoff event (Fig. 6b). The concentration of RP (<0.45) was lower than TP (<0.45) for the whole duration of the peak. In the peak event on 28 June 2011, on average 8.7 % of the P concentration was UP (<0.45) (Fig. 6b). Similar concentration differences were recorded in the other peak events. Observations in the arable catchment were too few to allow detection of any seasonal trend.

## Discussion

### Seasonality and pathways of P

In the forest catchment, the concentrations of TP (<0.45) showed a clear seasonal trend in both base flow and peak flow in the stream (Figs. 2, 3). Zelazny and Siwek (2012) reported a similar trend for a woodland catchment in Poland and attributed this higher TP (<0.45) concentration to catchment in-stream processes (Mulholland 1992). Soil water and groundwater TP (<0.45) concentrations remained low in our forest catchment, and hence could not have led to the high stream water concentrations during base flow conditions (Fig. 2). Point sources of P were also ruled out in this catchment. During the year, much vegetative material falls into the stream, and in summer, when temperatures are high, microbial breakdown of those compounds could have raised the TP (<0.45) concentration in the stream (Pozo et al. 2011). The decrease in concentration during peak flows (Fig. 4b) supports this assumption. Van Gaelen et al. (2014) conducted an end-member mixing analysis in this catchment and found that during base flow, 85 % of the water is contributed by the groundwater. Because the groundwater contains low concentrations of TP (<0.45), the high TP (<0.45) concentrations in the stream water during base flow conditions point to in-stream formation of P. During peak flow, the highest contribution to water flow originates from precipitation falling through the forest canopy (50 %), while only 15 % comes from the riparian zone as subsurface throughflow (Van Gaelen et al. 2014). The fact that the maximum contribution from the riparian zone comes only after the peak discharge (Van Gaelen et al. 2014) may explain the small increase in concentration that we observed after the peak (Fig. 4b). Riparian zones are often described as sources or sinks of nutrients in forest catchments (Mulholland 1992). They can hold an internal source of P and this internal source can be flushed out in rainfall events or mobilised by redox conditions.

In the pasture catchment, variations in stream water P concentrations differed from those in the forest catchment. Pasture catchment peak flows contained higher concentrations of TP (<0.45) than base flow samples. According to Van Gaelen et al. (2014), seeps contribute almost 100 % of the base flow discharge in the stream. Even during peak flows, the seeps contribute on average 74 and 81 % of the flow in spring–summer and autumn–winter peak flows, respectively. This means that the P concentration in those seeps largely determines the concentrations of P in the stream. Only during peak discharges another source of P becomes active. In the pasture catchment, these contributions can come from overland flow and subsequent dissolution of cow manure or from subsurface flow (Dougherty et al. 2004). The pasture is periodically grazed by cattle, supporting this assumption. The occasionally high concentration of TP (<0.45) in the suction cups indicates the presence of preferential flow paths that contain a high concentration of P infiltrating from surface sources (e.g. Stamm et al. 1998; Heathwaite and Dils 2000). In the pasture catchment, it is likely that the higher P concentrations during the peak flows come from saturation excess runoff in the riparian zone close to the river. These peak flows contribute on average 32 % of the yearly P export.

In the arable catchment, the only pathway by which P can enter the stream is through erosion of particles and dissolution of P into overland flow generated during a storm. Because discharge in the stream is only generated as overland flow from adjacent land, sources from riparian zones and preferential flow are negligible. Overland flow-generated erosion is known to be the main cause of P transfer in hilly arable areas prone to erosion (McDowell et al. 2001). The concentration of RP (<0.45) is curvilinearly dependent on the soil P status of arable land (Wang et al. 2010). The small amount of UP (<0.45) (8.7 %) present in the runoff from the arable catchment studied here can be attributed to the use of organic fertiliser on arable land (Ebeling et al. 2002), or to desorption from the colloidal fraction of the eroded soil material. Grassed waterways are used as a buffer zone against erosion of sediments in this area, but the concentration of P (<0.45) in stream water is still high. It is known that grassed waterways can be effective in trapping eroded sediment and related particulate P, but they are not effective in trapping the dissolved fraction of P. Moreover, higher export of TP (<0.45) can be expected if the trapped sediment loses P to the overflowing water in the long term (Hoffmann et al. 2009).

### Comparing P fluxes under different land uses

In order to estimate and compare areal fluxes of TP (<0.45) from river catchments, these have to be hydrologically closed. The pasture catchment is hydrologically closed, as the average difference between measured annual precipitation and stream discharge amounted to 506 mm year^−1^ (Table 2), which is the typical annual evapotranspiration from pasture in Flanders (Batelaan and De Smedt 2007). For the arable catchment, the difference between measured annual precipitation (632 mm, Table 2) and typical annual evapotranspiration (500 mm; Batelaan and De Smedt 2007) amounted to 132 mm, much more than the observed average annual stream flow of 26 mm. The missing 106 mm year^−1^ in the water balance gives an estimate of groundwater flow across the catchment boundaries. Using the average TP (<0.45) concentration of 0.045 mg P L^−1^ measured in a Flemish Environment Agency (VMM) monitoring well in the phreatic aquifer within the catchment (Databank Ondergrond Vlaanderen 2014), TP (<0.45) exports via the groundwater were an estimated 0.06 kg P ha^−1^, which is less than the 0.10–0.22 kg TP (<0.45) ha^−1^ exported as stream discharge comprising runoff only. When the same calculation procedure was used for the forest catchment, the TP (<0.45) flux out of the catchment via groundwater flow was 0.017 kg TP (<0.45) ha^−1^ year^−1^, compared with average export in stream flow of 0.0018 kg TP (<0.45) ha^−1^ year^−1^ (range 0.0014–0.0022; Table 2). Exports through groundwater flow may thus constitute a large proportion (90 %) of the TP (<0.45) exports from the forested catchment. However, even when accounting for this missing pathway, the total TP (<0.45) exports from the forested catchment were still 11-fold smaller than the exports from the arable catchment, where most losses occurred via runoff.

While land use was presumably the dominant factor in causing the differences in TP (<0.45) exports, differences in topography and hydrogeology may also have played a role. These factors are difficult to separate, because arable land is usually located in flatter areas, while forest and pasture are more common on steeper or wetter areas. However, it is clear that agricultural practices alter processes such as runoff and erosion and therefore exacerbate the inherent risk of P losses. Arable and pasture land also receive high inputs of P through fertiliser and animal manure, in turn elevating the risk of P losses to surface waters.

## Conclusions

Determination of P concentrations in environmental samples is very important when setting environmental limits to reduce eutrophication in rivers. Intensive monitoring of three headwater catchments under contrasting land uses revealed that annual exports of dissolved P were 0.08–0.28 kg P ha^−1^ year^−1^ from arable land and pasture and 0.021–0.022 kg P ha^−1^ year^−1^ from forest, including the losses via disconnected groundwater. Thus, agricultural practices can elevate areal P losses by up to 11-fold compared with the natural P losses in a forest catchment. This is because agricultural land receives high inputs of external P, including animal manure. These external inputs of manure did not elevate the DOP concentration, which was negligible in the forest and pasture catchment and small (8.7 % of dissolved TP) in the arable catchment. However, processes in the forest catchment occasionally caused elevated concentrations of P in headwater streams (up to 0.64 mg P L^−1^), for example rapid breakdown of leaf litter in summer due to high temperatures and high microbial activity increased P concentrations in headwater streams to far above environmental limits. In the forest catchment, high concentrations of P in base flow were most likely caused by in-stream processes. In the pasture catchment, TP (<0.45) concentrations increased during peak flow to well above the combined concentrations in groundwater, soil water and seep water. This indicates that processes such as runoff and subsurface flow, which contain higher concentrations of P, cause this rise. In the arable catchment, surface runoff is the main factor causing export of dissolved P, with high rainfall and subsequent overland flow exporting a high amount of TP (<0.45) (0.10–0.22 kg ha^−1^ year^−1^) in just a few (3–6) events. Losses of dissolved forms of P are difficult to reduce after dissolution of P in runoff water has occurred.

Agricultural practices exert pressures on surface waters. These need to be reduced by implementing an appropriate policy that also covers hydrological factors causing P to be transported from soil to surface waters.
